# Robotic Use, Operative Length, and Readmission after Duodenal Switch: A Retrospective Ten-Year MBSAQIP Analysis Using an Interaction Model

**DOI:** 10.1007/s11695-026-08921-w

**Published:** 2026-09-01

**Authors:** Samuel Leslie, Sruthi Shankar, Jordan Sauve, Amogh Kalyanam, Sayeed Ikramuddin, Eric Wise

**Affiliations:** 1 Dartmouth College, Hanover, USA; 2 Department of Surgery, University of Minnesota, Minneapolis, USA

**Keywords:** Duodenal switch, Robotic surgery, Class II and higher obesity, Readmission

## Abstract

**Background:**

Robotic-assisted surgery (RAS) is increasingly used in bariatric practice, yet its impact on outcomes after duodenal switch (DS) remains poorly characterized, and whether operative length (OL) modifies the RAS-outcome association has not been assessed. The present study examines the independent and interactive effects of RAS and OL on 30-day readmission after DS using a national cohort of over 17,000 cases from the MBSAQIP database spanning 2015–2024.

**Methods:**

We analyzed MBSAQIP data from 2015 to 2024 for patients undergoing laparoscopic DS (CPT 43845), excluding open, endoscopic, hand-assisted, and single-incision, revision, and conversion cases. Multivariable logistic regression assessed predictors of 30-day readmission, with a pre-specified interaction term between RAS and standardized OL to isolate the independent contribution of robotic use.

**Results:**

Among 17,615 DS cases, 5,496 (31.2%) were robotic-assisted. Thirty-day readmission was higher after RAS than laparoscopic DS (6.2% vs. 5.0%, *p* < 0.01). In adjusted models, RAS (OR 1.78, 95% CI 1.25–2.55, *p* < 0.05) and OL (OR 1.27 per standard deviation (SD), 95% CI 1.18–1.38, *p* < 0.001) were independently associated with increased odds of readmission. The RAS–OL interaction significantly improved model fit (likelihood ratio *p* = 0.007); each SD decrease in OL increased the relative readmission risk for RAS versus laparoscopic cases (interaction OR 0.79, 95% CI 0.67–0.94, *p* < 0.05). Temporal subgroup analysis shows this disadvantage was concentrated in 2015–2019.

**Conclusions:**

RAS for DS is associated with higher 30-day readmission, and OL significantly modifies this relationship. The interaction term suggests that improved operative efficiency will not resolve the RAS readmission disadvantage.

**Supplementary Information:**

The online version contains supplementary material available at https://doi.org/10.1007/s11695-026-08921-w.

## Introduction

Robotic-assisted surgery (RAS) has expanded over the past three decades, paralleling the wider adoption of minimally invasive surgical techniques. However, the evidence base for RAS in Duodenal Switch (DS), a more complex and less commonly performed procedure remains limited compared with sleeve gastrectomy (SG) and Roux-en-Y gastric bypass (RYGB) [1]. Prior DS literature has consisted largely of single-center studies or procedure-specific technical reports. One particularly relevant high volume single-center robotic DS study found that over a study period robotic cases operation length decreased from 187 to 139 min and length of stay fell from 3.4 to 2.2 days, showing improved operative times and outcomes with experience. Of the national database studies comparing RAS to laparoscopic DS, there have been conflicting results—some reporting lower readmission with RAS (*p* < 0.01) [2] and a more recent study reporting higher readmission and morbidity associated with RAS (odds ratio [OR] = 1.34, 95% confidence interval [CI] = 1.16–1.56, *p* < 0.001) [3]. Operative times have remained consistently longer with RAS, with mean durations of 198 versus 134 min reported for robotic and laparoscopic DS, respectively [3], though single-center data suggest this gap narrows with increasing experience [4]. Despite signals from national database analyses that RAS may be associated with higher readmission in DS, whether operative length independently modifies this relationship- rather than simply confounding it- has not been examined.

A key methodological limitation in prior bariatric literature is the treatment of operative length (OL) solely as a covariate rather than as a potential effect modifier of the relationship between RAS and readmission. Consequently, the independent contribution of the robotic platform may be obscured by differences in operative length across techniques and along the learning curve, making it difficult to isolate whether observed outcome differences reflect platform-specific risk or simply longer operative times. The present study addresses this gap by examining the independent and interactive effects of RAS and operative length on 30-day readmission after DS using a national cohort of over 17,000 cases from the MBSAQIP database spanning 2015–2024.

We leveraged the Metabolic and Bariatric Surgery Accreditation and Quality Improvement Program (MBSAQIP) to evaluate whether RAS is associated with 30-day readmission after DS and to test whether OL modifies this association. We prespecified an interaction between RAS and standardized OL to isolate the platform effect beyond time in the operating room, addressing an unmet need in MBS outcomes research.

## Methods

### Study Design and Data Source

We conducted a retrospective observational cohort study using the American College of Surgeons Metabolic Bariatric Surgery Accreditation Quality Improvement Project (ACS-MBSAQIP) participant use files from 2015 to 2024. The database contains standardized, clinically abstracted data on metabolic and bariatric operations performed at MBSAQIP-accredited centers in the United States. This study does not require institutional review board (IRB) consent because of the de-identified nature of the participants in this data set.

### Study Population

**Fig. 1:**
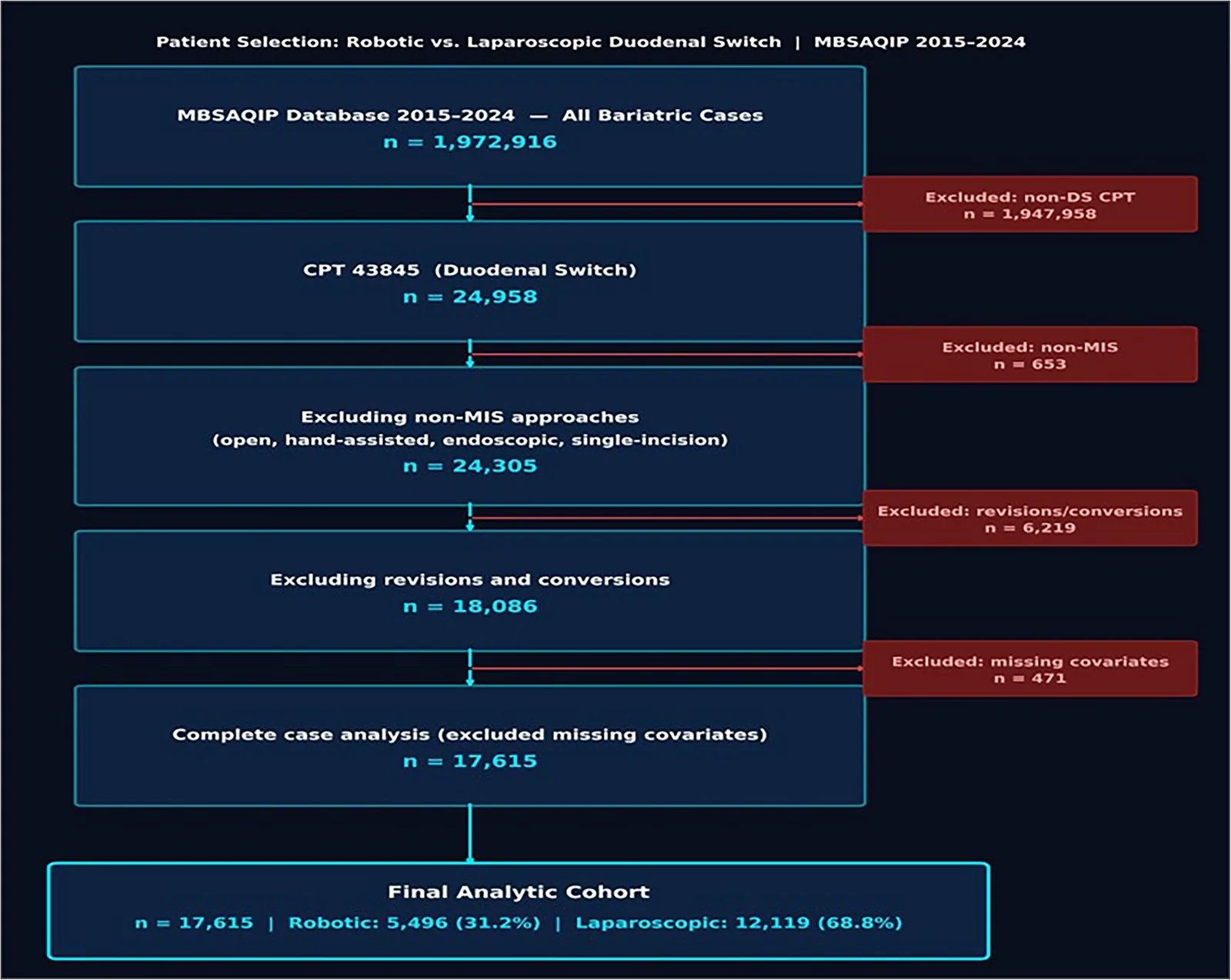
Flow diagram showing patient selection from 2015–2024 MBSAQIP database

We identified adult patients who underwent duodenal switch using CPT 43845. We restricted the cohort to minimally invasive approaches and excluded cases coded as open, endoscopic, hand-assisted, or single-incision procedures. We also excluded cases labeled as revisions or conversions to focus on primary DS procedures. Cohort assembly and final counts are reported in the Results (Fig. 1).

### Exposure and Comparator

The exposure of interest was robotic‑assisted surgery (RAS) versus laparoscopic (non‑robotic) DS, as captured by MBSAQIP approach variables. We use RAS to denote any case performed with a robotic platform and laparoscopic to denote conventional non‑robotic minimally invasive DS.

### Outcome

The primary outcome was 30‑day readmission, defined per MBSAQIP standard protocol. Readmission was selected a priori for its clinical and economic relevance and its standardized capture within the 30‑day follow‑up window.

### Covariates

Covariates included demographics (age, sex, race) and baseline body mass index (BMI). Comorbidities: diabetes, smoking status, chronic obstructive pulmonary disease (COPD), history of pulmonary embolism (PE), sleep apnea, gastroesophageal reflux disease (GERD), prior foregut surgery, history of myocardial infarction (MI), prior percutaneous coronary intervention (PCI), prior cardiac surgery, therapeutic anticoagulation, history of venous thrombosis, hyperlipidemia, renal insufficiency, and dialysis. Operative factors: operative length (OL, minutes) and operation year. To maintain consistency, we use the term operative length (OL) throughout.

### Statistical Analysis

Baseline characteristics were compared between RAS and laparoscopic cohorts using Welch’s t-test for continuous variables and chi-squared tests for categorical variables. A threshold of *p* < 0.01 denoted statistical significance to mitigate type I error from multiple comparisons.

For the primary analysis, we fit a multivariable logistic regression with 30‑day readmission as the dependent variable. Continuous variables were standardized (z‑scored) so that odds ratios (ORs) for continuous predictors (e.g., OL) represent the effect per 1 standard deviation (SD), facilitating comparison of effect sizes across predictors measured on different scales. The model included the covariates listed above and two prespecified terms of interest: RAS (yes/no) and OL (per SD).

To test whether OL modifies the association between RAS and readmission, we added a prespecified interaction term (RAS × standardized OL). Additionally, the linearity of the log odds for operation length were verified with display of differing slopes. Similar approaches Model fit for specifications with versus without the interaction was compared using a likelihood ratio test (LRT). We interpreted individual regression coefficients, including the interaction, at α = 0.05. Adjusted odds ratios (ORs) with 95% confidence intervals (CIs) are reported. Annual trends in RAS utilization are presented descriptively.

### Missing Data

We performed complete-case analysis for descriptive and multivariable models. Observations with missing fields required for a given analysis were excluded from that analysis only. No variable exhibited missingness prohibitive to model convergence.

### Sensitivity and Secondary Analyses (Prespecified)

Planned sensitivity analyses included:

1. Model without standardization of continuous predictors;

2. Model excluding ‘prior surgery’;

3. BMI subgroup analysis (≥ 50 kg/m^2^);

4. Propensity score weighting (IPTW) for RAS, contingent on acceptable covariate balance diagnostics.

5. Temporal subgroup analysis, splitting into an earlier era (2015–2019) and a recent era (2022–2024), to assess whether the RAS-OL interaction is stable over time.

Propensity score weighting was used to reduce confounding by indication inherent to the observational design [5]. Execution and results of these analyses are reported in the Results.

### Ethical Considerations

MBSAQIP data are de-identified and collected under standardized ACS protocols. Per institutional policy, this analysis of de-identified data was determined exempt / non-human subjects research. The ACS-MBSAQIP and participating centers are not responsible for the analyses or conclusions.

## Results

### Participants

From the ACS-MBSAQIP database, a total of 17,615 patients underwent minimally invasive DS and met inclusion criteria as seen in the patient flow diagram in Fig. 1. Of these, 5,496 (31.2%) were performed using RAS, while 12,119 (68.8%) used a conventional, laparoscopic approach.

### Baseline Characteristics

Baseline, demographic, clinical, and operative characteristics by surgical approach are shown in Table 1. Patients undergoing RAS were younger and had a higher baseline body mass index (BMI) compared with laparoscopic cases (*p* < 0.01 for both). RAS cases were also associated with a longer operative length and were performed in more recent calendar years (*p* < 0.01). Several comorbidities differ between groups. Patients in the RAS cohort more frequently had a history of prior surgery and preoperative vein thrombosis, whereas gastric disease and previous foregut surgery was more prevalent among laparoscopic cases (*p* < 0.001). Other comorbid conditions, including diabetes, smoking status, sleep apnea, and hyperlipidemia, were similar between groups. Robotic DS cases were associated with higher 30-day readmission (6.2% vs. 5.0%, *p* < 0.01).

**Table 1:** Baseline demographic, clinical, and operative characteristics of patients undergoing duodenal switch by surgical approach, MBSAQIP 2015–2024

Variable	Laparoscopic (*n* = 12,119)	Robotic-Assisted (*n* = 5,496)
**Age (years, SD)**	**43.40 ± 11.38**	**42.25 ± 10.69**
**Operation Length (min)**	**122.75 ± 63.77**	**177.57 ± 71.70**
**BMI**	**52.69 ± 9.59**	**55.13 ± 9.96**
**Operation Year**	**2019.79 ± 2.65**	**2020.92 ± 2.55**
Female Sex	8935 (73.7%)	4121 (75.0%)
**Race/ethnic minority**	**1667 (13.8%)**	**1331 (24.2%)**
Diabetes	3997 (33.0%)	1736 (31.6%)
**30-Day Readmission**	**609 (5.0%)**	**340 (6.2%)**
30-Day Reoperation	315 (2.6%)	138 (2.5%)
Smoker	1012 (8.4%)	415 (7.6%)
Chronic Obstructive Pulmonary Disease	227 (1.9%)	74 (1.3%)
History of Pulmonary Embolism	208 (1.7%)	86 (1.6%)
Sleep Apnea	5503 (45.4%)	2400 (43.7%)
**Gastroesophageal Reflux Disease**	**3**,**603 (29.7%)**	**1**,**389 (25.3%)**
**Prior Foregut Surgery**	**255 (2.1%)**	**49 (0.9%)**
History of Myocardial Infarction	170 (1.4%)	65 (1.2%)
Previous PCI	212 (1.7%)	78 (1.4%)
Previous Cardiac Surgery	94 (0.8%)	29 (0.5%)
Therapeutic Anticoagulation	429 (3.5%)	191 (3.5%)
Prior Venous Thrombosis	262 (2.2%)	122 (2.2%)
Hyperlipidemia	3151 (26.0%)	1378 (25.1%)
Renal Insufficiency	87 (0.7%)	35 (0.6%)
Dialysis	29 (0.2%)	9 (0.2%)

Data presented as mean ± SD or n (%). Bold highlights *p* < 0.01. BMI body mass index (kg/m²)

### Outcomes

The likelihood ratio test using the interaction term resulted in a p-value = 0.007; therefore, this complex model provides a statistically significant improvement in fit over the model without the interaction term. OL (odds ratio [OR] and 95% CI:1.27 [1.18, 1.38]) and robotic use (OR and 95% CI: 1.78 [1.25, 2.55]) predicted higher odds of readmission. Gastroesophageal reflux disease (OR and 95% CI: 1.24 [1.07, 1.43]) and therapeutic anticoagulation (OR and 95% CI: 1.6 [1.17, 2.19]) were also associated with increased odds of 30-day readmission (Table 2). Odds of readmission decrease by 7% in each year of the dataset (OR and 95% CI: 0.93 [0.91, 0.95]). Without the interaction term, RAS was not significant (OR, 95% CI, and P-value: 1.15 [0.98, 1.33], *p* = 0.08). Use of the robot in DS cases is associated with a 78% higher chance of 30-day readmission in comparison to the laparoscopic technique (OR and CI: 1.78 [1.25, 2.55]). Increased OL also has a 27% higher odds of 30-day readmission regardless of technique (OR and CI: 1.27 [1.18, 1.38] for each standard deviation (SD) increase (one SD for operation length is approximately 71 min).

**Table 2:** Multivariable logistic regression analysis of predictors of 30-day readmission following duodenal switch surgery

Independent Variables	Odds Ratio [95% CI]	*P*-value
**Robotic-Assisted Surgery (RAS)**	**1.78 [1.25**,** 2.55]**	**< 0.05**
Age	1.03 [0.96, 1.11]	0.39
Diabetes	0.96 [0.83, 1.12]	0.62
**Operation Length**	**1.27 [1.18**,** 1.38]**	**< 0.001**
**Operation Year**	**0.93 [0.91**,** 0.95]**	**< 0.001**
**Interaction Term (RAS x Operation Length)**	**0.79 [0.67**,** 0.94]**	**< 0.05**
**Race/ethnic minority**	**1.3 [1.1**,** 1.54]**	**< 0.05**
Female Sex	1.07 [0.91, 1.24]	0.43
BMI	1.03 [0.96, 1.1]	0.47
**Therapeutic Anticoagulation**	**1.6 [1.17**,** 2.19]**	**< 0.05**
Prior Venous Thrombosis	0.99 [0.65, 1.51]	0.95
**Hyperlipidemia**	**1.22 [1.04**,** 1.45]**	**< 0.05**
**Renal Insufficiency**	**2.02 [1.13**,** 3.61]**	**< 0.05**
Dialysis	0.79 [0.22, 2.79]	0.71
Smoker	1.14 [0.91, 1.44]	0.26
Chronic Obstructive Pulmonary Disease	0.86 [0.53, 1.39]	0.53
History of Pulmonary Embolism	1.32 [0.84, 2.07]	0.23
Sleep Apnea	1.14 [0.99, 1.32]	0.07
**Gastroesophageal Reflux Disease**	**1.24 [1.07**,** 1.43]**	**< 0.05**
Prior Foregut Surgery	0.95 [0.57, 1.56]	0.83
History of Myocardial Infarction	0.67 [0.37, 1.23]	0.20
Previous PCI	1.35 [0.84, 2.15]	0.22
Previous Cardiac Surgery	1.39 [0.72, 2.67]	0.33

Bold indicates statistical significance (*p* < 0.05). OR odds ratio; CI confidence interval; RAS robotic-assisted surgery; BMI body mass index (kg/m²). Operation length modeled per one standard deviation increase. Operation year modeled per one calendar year increase

The interaction term indicates that each one SD increase in operative length reduces the relative readmission risk associated with RAS compared with laparoscopic surgery (OR and 95% CI: 0.79 [0.67, 0.94]), suggesting that the readmission disadvantage of RAS diminishes as operative length increases. Table 3; Fig. 1 demonstrate a steady rise in robotic utilization over time (with the exception of 2020), culminating in a 469% overall increase in robotic surgery cases (from 204 cases in 2015 to 957 cases in 2024).

**Table 3:** Annual trends in robotic-assisted duodenal switch, MBSAQIP 2015 to 2024

Year	Robotic Surgery	Total Cases
2015	22.72% (204)	898
2016	18.53% (199)	1,074
2017	17.54% (254)	1,448
2018	22.14% (438)	1,978
2019	24.44% (499)	2,042
2020	31.68% (421)	1,329
2021	33.38% (734)	2,199
2022	37.92% (917)	2,418
2023	38.01% (873)	2,297
2024	49.53% (957)	1,932

Robotic Surgery column presents the proportion and absolute volume of robotic-assisted cases per year. Total cases reflects all duodenal switch procedures in the MBSAQIP. 2024 data may reflect partial-year reporting

### Sensitivity Analyses

**Fig. 2:**
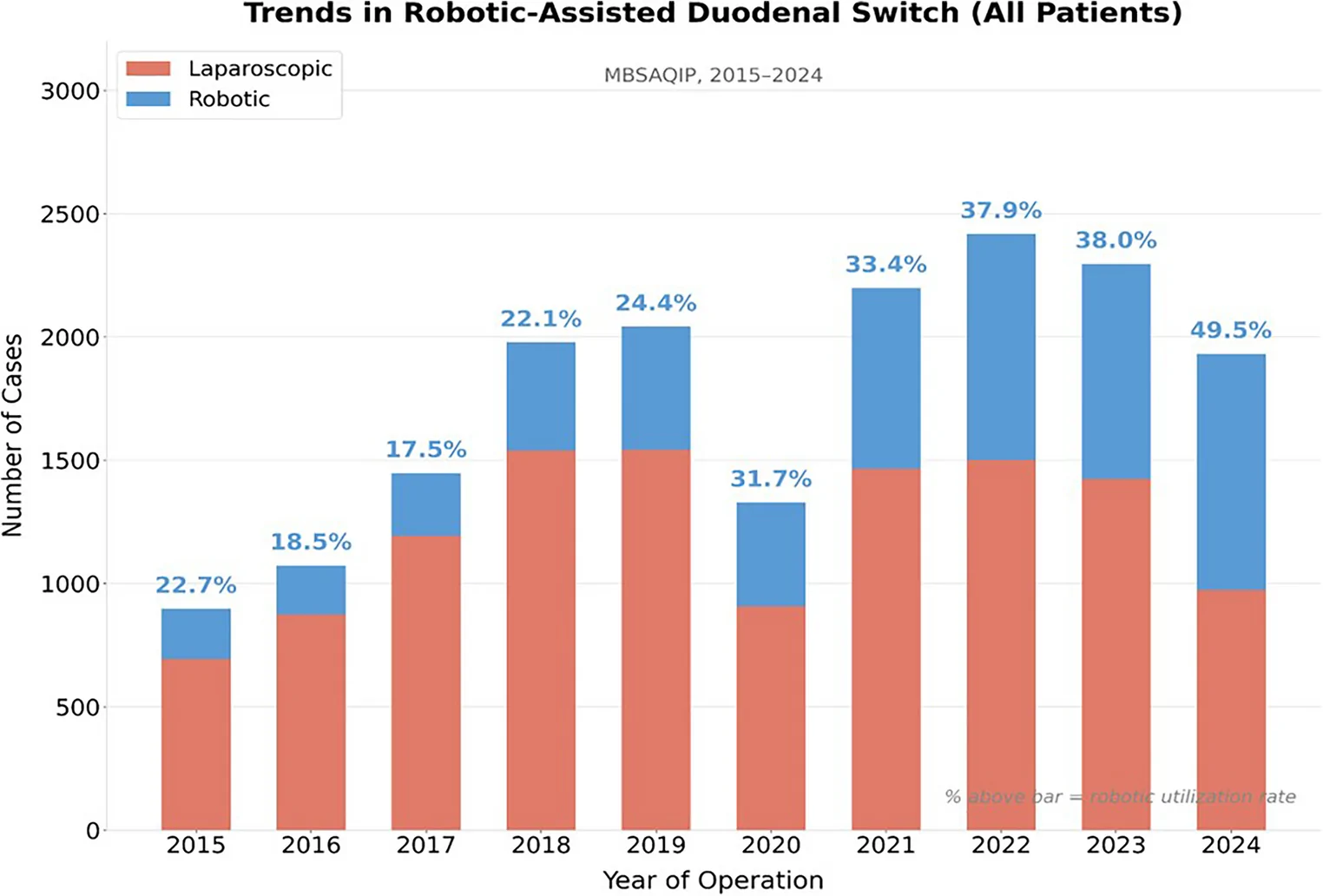
Trends in robotic-assisted approach for patients who underwent duodenal switch, MBSAQIP 2015–2024

The primary findings were consistent with sensitivity analyses. The model excluding previous foregut surgery resulted in an identical estimate (OR and CI: 1.78 [1.25, 2.55]), while the IPTW model, which balanced confounders between groups, confirmed that RAS remained independently associated with higher readmission, though the effect size was smaller (OR and CI: 1.31 [1.2, 1.44]; Supplementary Tables 1 and Supplementary Table 2). In the BMI > 50 kg/m^2^ subgroup, RAS was not significantly associated with readmission (OR and CI: 1.19 [0.78, 1.83]), and the interaction term was not significant (OR and CI: 0.95 [0.77, 1.17]), indicating that the primary finding may not hold in higher-BMI patients (Supplementary Tables 3 and Supplementary Table 4). A temporal subgroup analysis (Tables 4 and 5) splitting the cohort into an earlier era (2015–2019; *n* = 7,440) and a recent era (2020–2024; *n* = 10,175) showed a lower point estimate and loss of statistical significance for RAS and for the interaction term in the recent era relative to the earlier era. In the earlier era, RAS was strongly associated with higher readmission odds (OR and CI: 3.70 [1.94, 7.06]) and a significant interaction term (OR and CI: 0.58 [0.43, 0.79]). In the recent era, neither RAS (OR and CI: 1.40 [0.89, 2.19]) nor the interaction term (OR and CI: 0.87 [0.69, 1.08]) remained significant. A formal test of an era-by-RAS interaction was not performed, so this difference in significance across eras should not itself be interpreted as evidence that the underlying effect changed (Fig. 2).

**Table 4:** Multivariable logistic regression analysis of predictors of 30-day readmission following duodenal switch surgery, earlier era (2015–2019)

Independent Variables	Odds Ratio [95% CI]	*P*-value
**Robotic-Assisted Surgery**	**3.7 [1.94**,** 7.06]**	**< 0.001**
Age	0.97 [0.87, 1.08]	0.52
Diabetes	0.99 [0.79, 1.23]	0.89
**Operation Length**	**1.23 [1.1**,** 1.38]**	**< 0.001**
**Interaction Term (RAS x Operation Length)**	**0.58 [0.43**,** 0.79]**	**< 0.001**
**Race (Minority)**	**1.42 [1.1**,** 1.83]**	**< 0.05**
Female Sex	1.18 [0.94, 1.47]	0.16
BMI	1.0 [0.91, 1.11]	0.93
Therapeutic Anticoagulation	1.51 [0.95, 2.4]	0.08
Preoperative Vein Thrombosis	1.12 [0.62, 2.02]	0.70
Hyperlipidemia	1.19 [0.93, 1.51]	0.17
Renal Insufficiency	1.58 [0.65, 3.84]	0.31
Dialysis	1.0 [0.2, 5.0]	1.00
Smoker	1.04 [0.77, 1.42]	0.78
Chronic Obstructive Pulmonary Disease	0.73 [0.38, 1.42]	0.35
History of Pulmonary Embolism	1.16 [0.61, 2.22]	0.66
Sleep Apnea	1.09 [0.89, 1.34]	0.41
**Gastroesophageal Reflux Disease**	**1.3 [1.06**,** 1.59]**	**< 0.05**
Previous Surgery	1.25 [0.7, 2.24]	0.45
History of Myocardial Infarction	0.72 [0.32, 1.59]	0.41
Previous PCI	1.85 [1.0, 3.44]	0.05
Previous Cardiac Surgery	1.14 [0.44, 2.94]	0.79

Bold indicates statistical significance (*p* < 0.05). OR odds ratio; CI confidence interval; RAS robotic-assisted surgery; BMI body mass index (kg/m²). Operation year was also included in the model but is omitted from the table above as its effect rounds to 1.0 at one decimal place given the five-year span of this subgroup

**Table 5:** Multivariable logistic regression analysis of predictors of 30-day readmission following duodenal switch surgery, recent era (2020–2024)

Independent Variables	Odds Ratio [95% CI]	*P*-value
Robotic-Assisted Surgery	1.4 [0.89, 2.19]	0.14
Age	1.11 [1.0, 1.23]	0.06
Diabetes	0.96 [0.77, 1.18]	0.68
**Operation Length**	**1.37 [1.23**,** 1.54]**	**< 0.001**
Interaction Term (RAS x Operation Length)	0.87 [0.69, 1.08]	0.21
Race (Minority)	1.2 [0.96, 1.49]	0.11
Female Sex	0.98 [0.79, 1.21]	0.83
BMI	1.05 [0.95, 1.15]	0.33
**Therapeutic Anticoagulation**	**1.66 [1.07**,** 2.55]**	**< 0.05**
Preoperative Vein Thrombosis	0.91 [0.49, 1.7]	0.78
Hyperlipidemia	1.25 [0.99, 1.57]	0.06
**Renal Insufficiency**	**2.67 [1.24**,** 5.75]**	**< 0.05**
Dialysis	0.54 [0.06, 4.59]	0.57
Smoker	1.23 [0.88, 1.74]	0.23
Chronic Obstructive Pulmonary Disease	1.18 [0.58, 2.38]	0.65
History of Pulmonary Embolism	1.44 [0.76, 2.73]	0.26
Sleep Apnea	1.18 [0.97, 1.44]	0.10
Gastroesophageal Reflux Disease	1.15 [0.94, 1.42]	0.18
Previous Surgery	0.48 [0.18, 1.33]	0.16
History of Myocardial Infarction	0.6 [0.23, 1.56]	0.30
Previous PCI	0.88 [0.42, 1.88]	0.75
Previous Cardiac Surgery	2.09 [0.83, 5.24]	0.12

Bold indicates statistical significance (*p* < 0.05). OR odds ratio; CI confidence interval; RAS robotic-assisted surgery; BMI body mass index (kg/m²). Operation year was also included in the model but is omitted from the table above as its effect rounds to 1.0 at one decimal place given the five-year span of this subgroup

## Discussion

In this national ten-year analysis of over 17,000 duodenal switch procedures, RAS was independently associated with increased odds of 30-day readmission compared with laparoscopic DS, an effect significantly modified by operative length. The interaction term indicates that the readmission risk disadvantage in RAS was most pronounced for shorter operative times and diminished as operative length increased, though RAS carried higher readmission odds than laparoscopic techniques across the full range of times. This is one of the first analyses to categorize the effect of robotics on bariatric surgery with emphasis on differential risks.

Prior studies comparing RAS and laparoscopic DS have largely reported comparable perioperative outcomes [6]. One national database analysis reported significantly lower readmission with RAS for DS after adjusting for operative time as a covariate [2]. However, that study examined whether operative length predicted readmission differently across approaches, a distinct question from whether operative length modifies the association between RAS and readmission itself. Without the interaction term, RAS in the present study similarly showed no statistically significant difference between the two approaches, consistent with this prior literature [7]. Studies that have focused on sleeve gastrectomy and roux-en y gastric bypass have tended to show higher complication and readmission rates for RAS compared to laparoscopic surgery [8]. It is only by accounting for operative length as an effect modifier that the independent readmission disadvantage of RAS in DS becomes apparent. Longer operative times have been independently associated with increased odds of complications, and this relationship is particularly relevant in the context of RAS, where operative times remain consistently longer than laparoscopic surgery—a pattern attributed to the need to master the robotic learning curve [9].

Whether mastery of the robotic learning curve ultimately closes the operative time gap remains uncertain. Despite a tenfold increase in annual robotic case volume from 2015 to 2023, robotic sleeve gastrectomy and roux-en y gastric bypass operative times remained significantly longer than laparoscopic in every year of MBSAQIP data, although the gap between the two decreased slightly over time, and were associated with higher rates of readmission and reoperation [10]. The interaction term in this study raises further doubt about whether operative efficiency gains will resolve the readmission disadvantage of RAS. Counterintuitively, the readmission penalty associated with RAS was most pronounced at shorter operative times and diminished as operative length increased, suggesting that risk may be inherent to RAS and that faster robotic cases will not necessarily translate into better outcomes relative to laparoscopic surgery. This challenges ideas that shorter operation lengths over time will lead to RAS usurping laparoscopic surgery in surgical outcomes [11].

In the BMI > 50 kg/m^2^ subgroup analysis, RAS was not significantly associated with readmission. This is consistent with prior literature suggesting that the robotic platform may confer technical advantages with super-obese patients, where enlarged liver, intra-abdominal fat, and a thick abdominal wall increase the difficulty of laparoscopic maneuverability [12]. At extreme BMI, the technical challenges of laparoscopy may diminish the platform difference [13], potentially explaining why the readmission disadvantage of RAS was not observed in this subgroup [14].

The temporal subgroup analysis further nuances this interpretation. The RAS readmission disadvantage was pronounced and highly significant in the early era (2015–2019) but attenuated to non-significance in the recent era (2020–2024), a shift not accompanied by any era-specific operative-length as reported elsewhere in this manuscript. Because the primary interaction model implies that shorter RAS operative times should preserve, not eliminate, relative readmission risk, this pattern is not fully explained by OL convergence alone. One possible explanation is accumulating platform experience beyond operative time itself; however, the MBSAQIP database does not capture surgeon- or center-level robotic case volume, so this cannot be tested directly with these data, and unmeasured secular changes in patient selection, technique, or perioperative care over the study period remain an equally plausible explanation [15]. This tempers the earlier suggestion that operative efficiency gains cannot resolve the RAS readmission disadvantage: while OL alone may be insufficient, unmeasured temporal factors, potentially including accumulating platform experience, may be attenuating the disadvantage over time, a possibility this dataset cannot directly confirm.

Although the MBSAQIP captures no cost or resource utilization data, it is important to consider cost implications of this study’s findings. Robotic surgery carries higher maintenance, acquisition, and operational costs: even before controlling for longer operative times and increased operating room utilization, a study found that robotic surgery added $3,000 more per case in total costs [16]—this tends to hold for bariatric surgery [17]. Given that bariatric-related readmissions are estimated to cost the healthcare system $234 million annually, the readmission disadvantage of RAS identified here, if persistent even as operative efficiency improves, makes the cost-benefit justification for widespread robotic adoption in duodenal switch increasingly difficult to support [3].

This study is limited by its observational status in which no causal relationships can be inferred. The MBSAQIP database excludes non-accredited MBSAQIP centers, limiting national representativeness, and does not capture surgeon-specific robotic experience, a critical confounder in interpreting platform-specific outcome differences, as surgeons at varying points on the robotic learning curve may contribute differentially to observed readmission rates. The 30-day readmission window precludes assessment of longer-term outcomes, and the exclusion of outpatient surgery may introduce selection bias. Cost and resource utilization data are absent from the MBSAQIP, limiting direct economic analysis. Randomized control trials remain necessary to fully account for systematic differences between robotic and laparoscopic patient populations and to establish causal relationships between surgical approach and postoperative outcomes [18].

## Conclusion

Compared to laparoscopic techniques in the full ten-year cohort, an effect significantly modified by operative length. Temporal subgroup analysis indicates that this disadvantage was concentrated in the early years of robotic adoption (2015–2019) and attenuated to non-significance in the most recent years (2020–2024). This difference in significance across eras was not formally tested and may partly reflect reduced power in the smaller early-era robotic subgroup. These findings suggest that while operative length alone does not fully explain the RAS readmission disadvantage, unmeasured factors, potentially including accumulating platform experience, which this dataset cannot directly measure, may be narrowing it over time. Longer-term outcomes studies will be needed to determine whether this trend continues toward full convergence with laparoscopic outcomes [19].

## Supplementary Information

Supplementary Material 1.

Supplementary Material 2.

Supplementary Material 3.

Supplementary Material 4.

## Data Availability

All data supporting the findings in this study were derived from publicly available datasets, and will be made available by reasonable request to the corresponding author.
