# Supplementary material for: Robotic Use, Operative Length, and Readmission after Duodenal Switch: A Retrospective Ten-Year MBSAQIP Analysis Using an Interaction Model
Source: Obes Surg. 2026 Sep 1;36(10):5388–95. doi: 10.1007/s11695-026-08921-w (PMC13645967; doi:10.1007/s11695-026-08921-w)
Supplement: Supplementary file 1 [file 11695_2026_8921_MOESM1_ESM.docx]

## **Supplementary Table 1:** Covariate balance before and after inverse probability of treatment weighting (IPTW).

| **Variable** | **SMD Before** | **SMD After** | **Balanced** |
| --- | --- | --- | --- |
| Age | 0.104 | 0.012 | Yes |
| Diabetes | 0.03 | 0 | Yes |
| Operation Year | 0.435 | 0.002 | Yes |
| Race (Minority) | 0.269 | 0.01 | Yes |
| Female Sex | 0.029 | 0.001 | Yes |
| BMI | 0.25 | 0.008 | Yes |
| Therapeutic Anticoagulation | 0.004 | 0.003 | Yes |
| Prior Venous Thrombosis | 0.004 | 0.004 | Yes |
| Hyperlipidemia | 0.021 | 0.008 | Yes |
| Renal Insufficiency | 0.01 | 0.003 | Yes |
| Dialysis | 0.017 | 0.008 | Yes |
| Smoker | 0.03 | 0.009 | Yes |
| Chronic Obstructive Pulmonary Disease | 0.042 | 0.018 | Yes |
| History of Pulmonary Embolism | 0.012 | 0.003 | Yes |
| Sleep Apnea | 0.035 | 0 | Yes |
| Gastroesophageal Reflux Disease | 0.1 | 0.024 | Yes |
| Prior Foregut Surgery | 0.1 | 0.026 | Yes |
| History of Myocardial Infarction | 0.019 | 0.002 | Yes |
| Previous PCI | 0.026 | 0.005 | Yes |
| Previous Cardiac Surgery | 0.031 | 0.005 | Yes |

Legend: SMD standardized mean difference. SMD < 0.10 indicates acceptable covariate balance. Balanced column indicates whether SMD after weighting met this threshold.
