# Supplementary material for: Robotic Use, Operative Length, and Readmission after Duodenal Switch: A Retrospective Ten-Year MBSAQIP Analysis Using an Interaction Model
Source: Obes Surg. 2026 Sep 1;36(10):5388–95. doi: 10.1007/s11695-026-08921-w (PMC13645967; doi:10.1007/s11695-026-08921-w)
Supplement: Supplementary file 2 [file 11695_2026_8921_MOESM2_ESM.docx]

## **Supplementary Table 2:** IPTW-weighted logistic regression analysis of 30-day readmission following robotic-assisted versus laparoscopic duodenal switch.

| **Independent Variables** | **Odds Ratio [95% CI]** | **P-value** |
| --- | --- | --- |
| **Robotic-Assisted Surgery** | **1.31 [1.2, 1.44]** | **<0.001** |
| Age | 1.04 [0.99, 1.1] | 0.10 |
| Diabetes | 0.96 [0.87, 1.07] | 0.49 |
| **Operation Year** | **0.93 [0.91, 0.94]** | **<0.001** |
| **Race (Minority)** | **1.41 [1.26, 1.58]** | **<0.001** |
| Female Sex | 1.04 [0.93, 1.16] | 0.47 |
| **BMI** | **1.06 [1.01, 1.11]** | **<0.05** |
| **Therapeutic Anticoagulation** | **1.56 [1.25, 1.95]** | **<0.001** |
| Prior Venous Thrombosis | 1.0 [0.75, 1.34] | 0.99 |
| **Hyperlipidemia** | **1.2 [1.07, 1.35]** | **<0.05** |
| **Renal Insufficiency** | **2.22 [1.48, 3.32]** | **<0.001** |
| Dialysis | 0.41 [0.13, 1.24] | 0.11 |
| **Smoker** | **1.2 [1.03, 1.4]** | **<0.05** |
| Chronic Obstructive Pulmonary Disease | 1.14 [0.84, 1.57] | 0.40 |
| **History of Pulmonary Embolism** | **1.44 [1.06, 1.96]** | **<0.05** |
| **Sleep Apnea** | **1.18 [1.07, 1.3]** | **<0.05** |
| **Gastroesophageal Reflux Disease** | **1.2 [1.09, 1.33]** | **<0.001** |
| Prior Foregut Surgery | 0.67 [0.43, 1.03] | 0.07 |
| History of Myocardial Infarction | 0.86 [0.58, 1.28] | 0.47 |
| Previous PCI | 1.29 [0.92, 1.81] | 0.14 |
| Previous Cardiac Surgery | 0.83 [0.49, 1.43] | 0.50 |

Legend: Bold indicates statistical significance (p < 0.05). OR odds ratio; CI confidence interval; IPTW inverse probability of treatment weighting; RAS robotic-assisted surgery; BMI body mass index (kg/m²). Operative length was excluded from this model as a post-treatment variable to avoid overadjustment bias
