# Supplementary material for: Robotic Use, Operative Length, and Readmission after Duodenal Switch: A Retrospective Ten-Year MBSAQIP Analysis Using an Interaction Model
Source: Obes Surg. 2026 Sep 1;36(10):5388–95. doi: 10.1007/s11695-026-08921-w (PMC13645967; doi:10.1007/s11695-026-08921-w)
Supplement: Supplementary file 3 [file 11695_2026_8921_MOESM3_ESM.docx]

## Supplementary Table 3: Baseline characteristics of patients with BMI ≥ 50 kg/m² undergoing duodenal switch by surgical approach, MBSAQIP 2015-2024

| **Variable** | **Laparoscopic (n=6,990)** | **Robotic-Assisted Surgery (n=3,747)** |
| --- | --- | --- |
| **Age** | **41.82 ± 10.92** | **41.02 ± 10.39** |
| **Operation Length (min)** | **130.77 ± 66.70** | **177.26 ± 68.15** |
| **BMI** | **58.82 ± 7.73** | **59.78 ± 8.46** |
| **Operation Year** | **2019.50 ± 2.61** | **2020.86 ± 2.57** |
| Female Sex | 5094 (72.9%) | 2798 (74.7%) |
| **Race/ethnic minority** | **997 (14.3%)** | **991 (26.4%)** |
| Diabetes | 2091 (29.9%) | 1044 (27.9%) |
| **30-Day Readmission** | **361 (5.2%)** | **244 (6.5%)** |
| 30-Day Reoperation | 193 (2.8%) | 92 (2.5%) |
| Smoker | 607 (8.7%) | 279 (7.4%) |
| Chronic Obstructive Pulmonary Disease | 130 (1.9%) | 58 (1.5%) |
| History of Pulmonary Embolism | 140 (2.0%) | 71 (1.9%) |
| Sleep Apnea | 3428 (49.0%) | 1767 (47.2%) |
| **Gastroesophageal Reflux Disease** | **1978 (28.3%)** | **918 (24.5%)** |
| **Prior Foregut Surgery** | **122 (1.7%)** | **32 (0.9%)** |
| History of Myocardial Infarction | 73 (1.0%) | 39 (1.0%) |
| Previous PCI | 82 (1.2%) | 37 (1.0%) |
| Previous Cardiac Surgery | 42 (0.6%) | 15 (0.4%) |
| Therapeutic Anticoagulation | 254 (3.6%) | 142 (3.8%) |
| Prior Venous Thrombosis | 165 (2.4%) | 87 (2.3%) |
| Hyperlipidemia | 1524 (21.8%) | 808 (21.6%) |
| Renal Insufficiency | 41 (0.6%) | 20 (0.5%) |
| Dialysis | 14 (0.2%) | 5 (0.1%) |

Legend: Data presented as mean ± SD or n (%). Bold indicates p < 0.01. BMI body mass index (kg/m²).
