# Supplementary material for: Robotic Use, Operative Length, and Readmission after Duodenal Switch: A Retrospective Ten-Year MBSAQIP Analysis Using an Interaction Model
Source: Obes Surg. 2026 Sep 1;36(10):5388–95. doi: 10.1007/s11695-026-08921-w (PMC13645967; doi:10.1007/s11695-026-08921-w)
Supplement: Supplementary file 4 [file 11695_2026_8921_MOESM4_ESM.docx]

## **Supplementary Table 4:** Multivariable logistic regression analysis of predictors of 30-day readmission rates in BMI ≥ 50 kg/m² subgroup.

| **Independent Variables** | **Odds Ratio [95% CI]** | **P-value** |
| --- | --- | --- |
| Robotic-Assisted Surgery | 1.19 [0.78, 1.83] | 0.42 |
| **Age** | **1.1 [1.0, 1.2]** | **<0.05** |
| Diabetes | 0.86 [0.71, 1.05] | 0.14 |
| **Operation Length** | **1.25 [1.13, 1.38]** | **<0.001** |
| **Operation Year** | **1.0 [1.0, 1.0]** | **<0.001** |
| Interaction Term (RAS x Operation Length) | 0.95 [0.77, 1.17] | 0.65 |
| **Race/ethnic minority** | **1.28 [1.05, 1.57]** | **<0.05** |
| **Female Sex** | **1.22 [1.0, 1.49]** | **<0.05** |
| BMI | 1.03 [0.95, 1.12] | 0.41 |
| Therapeutic Anticoagulation | 1.33 [0.88, 2.02] | 0.18 |
| Preoperative Vein Thrombosis | 0.87 [0.5, 1.51] | 0.63 |
| Hyperlipidemia | 1.15 [0.93, 1.43] | 0.21 |
| Renal Insufficiency | 0.4 [0.09, 1.84] | 0.24 |
| Dialysis | 1.64 [0.18, 14.67] | 0.66 |
| Smoker | 1.05 [0.78, 1.41] | 0.74 |
| Chronic Obstructive Pulmonary Disease | 1.16 [0.67, 2.02] | 0.59 |
| History of Pulmonary Embolism | 1.39 [0.81, 2.39] | 0.23 |
| **Sleep Apnea** | **1.23 [1.03, 1.46]** | **<0.05** |
| Gastroesophageal Reflux Disease | 1.19 [0.99, 1.43] | 0.06 |
| Prior Foregut Surgery | 0.72 [0.33, 1.56] | 0.41 |
| History of Myocardial Infarction | 0.97 [0.43, 2.17] | 0.93 |
| Previous PCI | 1.25 [0.6, 2.6] | 0.55 |
| Previous Cardiac Surgery | 0.75 [0.22, 2.53] | 0.65 |

Legend: Bold indicates statistical significance (p < 0.05). OR odds ratio; CI confidence interval; RAS robotic-assisted surgery; BMI body mass index (kg/m²). Operation length modeled per one standard deviation increase. Operation year modeled per one calendar year increase
